# *“Regardless, you are not the first woman”:* an illustrative case study of contextual risk factors impacting sexual and reproductive health and rights in Nicaragua

**DOI:** 10.1186/s12905-019-0771-9

**Published:** 2019-06-14

**Authors:** Samantha M. Luffy, Dabney P. Evans, Roger W. Rochat

**Affiliations:** Hubert Department of Global Health, Rollins School of Public Health, Emory University, 1518 Clifton Road, Mailstop: 1518-002-7BB, Atlanta, GA 30322 USA

**Keywords:** Nicaragua, Unsafe abortion, Sexual and reproductive rights, Unintended pregnancy, Violence against women

## Abstract

**Background:**

Rape, unintended pregnancy, and abortion are among the most controversial and stigmatized topics facing sexual and reproductive health researchers, advocates, and the public today. Over the past three decades, public health practicioners and human rights advocates have made great strides to advance our understanding of sexual and reproductive rights and how they should be protected. The overall aim of the study was to understand young women’s personal experiences of unintended pregnancy in the context of Nicaragua’s repressive legal and sociocultural landscape. Ten in-depth interviews (IDIs) were conducted with women ages 16–23 in a city in North Central Nicaragua, from June to July 2014.

**Case presentation:**

This case study focuses on the story of a 19-year-old Nicaraguan woman who was raped, became pregnant, and almost died from complications resulting from an unsafe abortion. Her case, detailed under the pseudonym Ana Maria, presents unique challenges related to the fulfillment of sexual and reproductive rights due to the restrictive social norms related to sexual health, ubiquitous violence against women (VAW) and the total ban on abortion in Nicaragua. The case also provides a useful lens through which to examine individual sexual and reproductive health (SRH) experiences, particularly those of rape, unintended pregnancy, and unsafe abortion; this in-depth analysis identifies the contextual risk factors that contributed to Ana Maria’s experience.

**Conclusions:**

Far too many women experience their sexuality in the context of individual and structural violence. Ana Maria’s case provides several important lessons for the realization of sexual and reproductive health and rights in countries with restrictive legal policies and conservative cultural norms around sexuality. Ana Maria’s experience demonstrates that an individual’s health decisions are not made in isolation, free from the influence of social norms and national laws. We present an overview of the key risk and contextual factors that contributed to Ana Maria’s experience of violence, unintended pregnancy, and unsafe abortion.

**Electronic supplementary material:**

The online version of this article (10.1186/s12905-019-0771-9) contains supplementary material, which is available to authorized users.

## Background

Rape, unintended pregnancy, and abortion are among the most controversial and stigmatized topics facing sexual and reproductive health researchers, advocates, and the public today. Over the past three decades, however, the international community, States, and advocates have made great strides to advance our understanding of sexual and reproductive rights and how they can be protected at the national and international levels. The 1994 Cairo Declaration began this process by including sexual health under the umbrella of reproductive health and recognized the impact of violence on an individual’s sexual and reproductive health (SRH) decision-making. [1] One year later, the 1995 Beijing Platform for Action specifically addressed the issues of unintended pregnancy and abortion by emphasizing that improved family planning services should be the main method by which unintended pregnancies and unsafe abortions are prevented. [2]

A recent World Health Organization (WHO) report on the relationships between sexual health, human rights, and State’s laws sets the foundation for our contemporary understanding of these issues. The 2015 report describes sexual health as, “a state of physical, emotional, mental and social well-being in relation to sexuality.” [3] That state includes control over one’s fertility via access to health services such as abortion; it also includes the right to enjoy sexual experiences free from coercion, discrimination, and violence. [3] Whether experienced alone or in combination, rape, unintended pregnancy, and abortion are important SRH issues on which public health can and should intervene.

In the public health field, case studies provide a useful lens through which to examine individual women’s sexual and reproductive health experiences, particularly those of rape, unintended pregnancy, and unsafe abortion; an in-depth analysis of these personal experiences can identify contextual risk factors and missed opportunities for public health rights-based  intervention. This type of analysis is especially cogent when legal policies and social factors, such as gender inequality, may influence one’s SRH decision-making process. On an individual level, bearing witness to women’s stories through in-depth interviews helps document their lived experience; surveying these experiences within the context of laws related to SRH provides important evidence for the impact of such policies on women’s well-being.

We present the case of a 19-year-old Nicaraguan woman who was raped, became pregnant, and almost died from complications resulting from an unsafe abortion. Her complex experience of violence, unintended pregnancy, and unsafe abortion represent a series of contextual factors and missed opportunities for public health and human rights intervention. Ana Maria’s story, told through the use of a pseudonym, takes place in a city located in North Central Nicaragua – a country that presents unique challenges related to its citizens’ fulfillment of their sexual and reproductive health and rights.

### Violence against women in Nicaragua

Along with 189 States, Nicaragua is a party to the United Nations (UN) Convention on the Elimination of All Forms of Discrimination against Women, which includes State obligations to protect and promote the health and well-being of Nicaraguan women. [4] As defined by human rights documents, the right to health includes access to health care services, as well as provisions for the underlying social determinants of health, such as personal experiences of structural violence. [5]

In the Nicaraguan context, political and sociocultural institutions support unequal power relations between genders. [6] *Machismo* is one such form of structural violence that perpetuates gender inequality and has been identified as a barrier to SRH promotion in Nicaragua. [7, 8] The term ‘*machismo*’ is most commonly used to describe male behaviors that are sexist, hyper masculine, chauvinistic, or violent towards women. [9] These behaviors often legitimize the patriarchy, reinforce traditional gender roles, and are used to limit or control the actions of women, who are often perceived as inferior. [10]

The vast majority (89.7%) of Nicaraguan women have experienced some form of gender-based violence  during their lifetime, which poses a serious public health problem. The latest population-based Demographic and Health Survey showed that at least 50% of Nicaraguan women surveyed had experienced either verbal/psychological, physical, or sexual violenceduring their lifetime. An additional 29.3% of women reported having experienced both physical and sexual violence at least once, while another 10.4% reported having experienced all three types of violence. [11]

In 2012, Nicaragua joined a host of other Central and South American countries that have implemented laws to eliminate all forms of violence against women VAW, including rape and femicide. [12] Nicaragua’s federal law against VAW, Law 779, intends to eradicate such violence in both public and private spheres. [13] On paper, Law 779 guarantees women freedom from violence and discrimination, but it is unclear if the law is being adequately enforced; it has been reported that some women believe VAW has increased since the law’s implementation. [14]

Before Law 779, violent acts like rape, particularly of young women ages 15–24, were endemic in Nicaragua. Approximately two-thirds of rapes reported in Nicaragua between 1998 and 2008 were committed against girls under 17 years of age; most of these acts were committed by a known acquaintance. [15] Due to a lack of reporting and to culturally propagated stigma regarding rape, no reliable data suggest that Law 779 has been effective in reducing the incidence of rape in Nicaragua. For women who wish to terminate a pregnancy that resulted from rape, access to abortion services is vital, yet completely illegal. [16] In contrast, technical guidance from the WHO recommends that health systems include access to safe abortion services for women who experience unintended pregnancy or become pregnant as a result of rape. [17]

### Family planning and unintended pregnancy in Nicaragua

Like violence, unintended pregnancies -- not only those that result from rape -- pose a widespread public health problem in Nicaragua. National data suggest that 65% of pregnancies among women ages 15–29 were unintended. [11] Oftentimes, unintended pregnancy results from a complex combination of social determinants of health including: low socioeconomic status (SES), low education level, lack of access to adequate reproductive health care, and restrictive reproductive rights laws. [18–20] Nicaraguan women of low SES with limited access to family planning services are at an increased risk of depression, violence, and unemployment due to an unintended pregnancy. [19, 20]

The UN Committee on the Elimination of all forms of Discrimination Against Women (CEDAW) has expressed concern regarding the lack of comprehensive sexual education programs, as well as inadequate family planning services, and high rates of unintended pregnancy throughout Nicaragua. [21] Due to a lack of sexual education, Nicaraguan adolescents, if they use contraceptives like male condoms or oral contraceptive pills, often do so inconsistently or incorrectly. [22]

Deeply rooted cultural stigma surrounding unmarried women’s sexual behavior contributes to the harsh criticism of young women in Nicaragua that use a method of family planning or engage in sexual relationships outside of a committed union. [18, 22] Also, young women who are not in a formal union may experience unplanned sex (consensual or nonconsensual) and are unlikely to be using contraception, which further increases the risk of unintended pregnancy. [22] These social and cultural factors, in conjunction with restrictive reproductive rights laws, may contribute to a high incidence of unintended pregnancy among young Nicaraguan women.

### The total ban on abortion in Nicaragua

Compounding the economic, social, and emotional burden of unintended pregnancy on women’s lives is the current prohibition of abortion in Nicaragua. In 2006, the National Assembly unanimously passed a law to criminalize abortion, which had been legal in Nicaragua since the late 1800s. [20] Researchers often refer to this law as the “total ban” on abortion. [20, 23] The total ban prohibits the termination of a pregnancy in all cases, including incest, rape, fetal anomaly, and danger to the life of the woman. Laws that prohibit medical procedures are, by definition, barriers to access; equitable access to safe medical services is a critical element of the right to health. [3, 5] The UN Committee on Civil and Political Rights (CCPR) has also recognized the discriminatory and harmful nature of criminalizing medical procedures that only women undergo. [24]

Nicaragua is one of the few countries in the world to completely ban abortion in all circumstances. In States where illegal, abortion does not stop. Instead, women are forced to obtain abortions from unskilled providers in conditions that are often unsafe and unhygienic. [25] Unsafe abortions are among the main preventable causes of maternal morbidity and mortality worldwide and can be avoided through decriminalization of such services. [26]

The Nicaraguan ban includes serious legal penalties for women who obtain illegal abortions, as well as for the medical professionals who perform them, which can have profound negative effects on women’s health. [20, 23] Women who need or want an abortion face not only the health risks that accompany an unsafe procedure, but additional criminal penalties. The total ban on abortion violates the human rights of both health care providers and women nationwide, as well as the confidentiality inherent in the patient-provider relationship. [20] It also results in a ‘chilling effect’ where health care providers are unwilling to provide both abortion and postabortion care (PAC) services for fear of prosecution. [20]

In response to the negative impacts of the total ban on maternal morbidity and mortality in Nicaragua, as well as detrimental effects on women’s physical, mental, and emotional health, CEDAW has recommended that the Nicaraguan government review the total ban and remove the punitive measures imposed on women who have abortions. [21] While the Nicaraguan government may not view abortion as a human right per se, women should not face morbidity or mortality as a result of illegal or unsafe abortion. [27]

Criminalizing abortion also increases stigma around this issue and significantly reduces people’s willingness to speak openly about abortion and related SRH services. Qualitative research conducted in Nicaragua suggests that women who have had unsafe abortions rarely discuss their experiences openly due to the illegal and highly stigmatized nature of such procedures. [18] Therefore, the overall aim of the study was to better understand young women’s personal experiences of unintended pregnancy in the context of Nicaragua’s repressive legal and sociocultural landscape. Ten in-depth interviews (IDIs) were conducted with women ages 16–23 in a city in North Central Nicaragua from June to July 2014. This private method of data collection allowed for the detailed exploration of each young woman’s personal experience with an unintended pregnancy, including the decision-making process she went through regarding how to respond to the pregnancy. Given the personal nature of this experience – including the criminalization and stigmatization of women who obtain abortions – IDIs allowed the participants to share intimate details and information that would be inappropriate or dangerous to share in a group setting. One case, presented here, emerged as salient for understanding the intersections of violence, unintended pregnancy, and abortion – and the missed opportunities for rights-based public health intervention.

Emory University’s Institutional Review Board ruled the study exempt from review because it did not meet the definition of “research” with human subjects as set forth in Emory policies and procedures and federal rules. Nevertheless, procedural steps were taken to protect the rights of participants and ensure confidentiality throughout data collection, management, and analysis. The first author reviewed the informed consent form in Spanish with each participant and then acquired each participant’s signature and verbal informed consent before the IDIs were conducted. The investigators developed a semi-structured interview guide with open-ended questions and piloted the guide twice to improve the cultural appropriateness of the script (Additional file 1). The investigators also collaborated with local partners to design and implement the research according to local cultural and social norms. Due to the contentious topics discussed in this study, these collaborators prefer to not be mentioned by name. Interviews were conducted in Spanish in a private location and audio taped to protect the participants’ privacy. Recordings were transcribed verbatim and transcripts were coded and analyzed using MAXQDA11 software (VERBI GmbH, Berlin, Germany).

Initially, participants were recruited for interviews through purposive sampling of individuals who had disclosed a personal experience with unintended pregnancy during focus group discussions (FGDs) conducted in a larger parent study. At the end of each interview, participants were asked to refer other young women they knew who may have experienced an unintended pregnancy to participate in an interview. This form of respondent-driven sampling created a network of participants with a wide variety of experiences with unintended pregnancy. Of the ten interviewees, two had experienced unintended pregnancy as a result of rape, though both used the phrase “*sexo no consensual*” or “nonconsensual sex” in lieu of “*violación,*” the Spanish word for rape. One of these women shared her personal experience receiving an unsafe abortion to terminate an unintended pregnancy that had resulted from rape. Her story, shared under the use of the pseudonym Ana Maria, is presented here in order to:

- Illustrate the harmful impact of restrictive abortion laws on the health and well-being of women – especially those who do not have access to abortion in the case of rape; and
- Exemplify the nexus of contextual risk factors that impact women’s SRH decision-making, such as conservative social norms and restrictive legal policies.

Through thorough analysis, we examine the impact of these contextual factors that impacted Ana Maria’s experience.

## Case presentation

When she was 19, Ana Maria was raped by her godfather, a close friend of her family.

In an in-depth interview, Ana Maria described enduring incessant verbal harassment from her godfather – her elder brother’s best friend – in the months before the assault. He constantly called and texted her cell phone in order to interrogate her about platonic relationships with other men in town and to convince her to spend time alone with him. Even though he was married with children and she repeatedly dismissed his advances, he continued to engage in this form of psychological violence with his goddaughter. Ana Maria described eventually “giving in” and meeting him – not knowing that this encounter would result in her forcible rape.

The disclosure of Ana Maria’s rape during her interview was spontaneous and unexpected. Ana Maria was unwilling to disclose explicit details of the sexual assault. Instead, she stated multiple times that the sexual contact was nonconsensual and she did not want to have sex with him. When asked if she told anyone about this experience, she said no because she did not want others to judge her for what had happened.

Approximately a month of scared silence after she was raped, Ana Maria noticed that her period had not come. Nervous, she bought a pregnancy test from a local pharmacy. To her dismay, the test was positive. In order to confirm the pregnancy, she traveled alone to the nearby health center in her town to obtain a blood test. Again, the test was positive. She had never been pregnant before and she was terrified. In the midst of her fear, she shared the results with her rapist, her godfather.

His response: get an abortion. He did not want to lose his wife and children if they found out about the pregnancy.

Other than their illegal nature, Ana Maria knew nothing about abortions – where to get one, how it was done, what it felt like. She asked her neighbors to explain it to her. They said “it was worse than having a baby and [experiencing] childbirth.”

Though Ana Maria did not want to get the abortion, her godfather continued to pressure her to get the procedure saying, “Regardless, you must get the abortion… you are not the first woman to have ever had one.” Similar to the emotional violence before he raped her, he called and texted Ana Maria every day telling her to, “do it as fast as you can.” He forbade her from telling anyone about the pregnancy and Ana Maria didn’t feel like she had anyone to confide in about the situation. She worried about people judging her for getting pregnant outside of a committed relationship – even though she was raped. Ana Maria described this difficult time:

“When he started to pressure me [to get the abortion], I felt alone. I did not have enough trust in anyone to tell them [what had happened] because… if I had had enough trust in someone, I know that they would not have let me do it. If I had been given advice, they would have said, ‘No, do not do it,’ but I did not have anyone and I felt so depressed. What made it worse, I couldn’t sleep; I could not sleep [because I was] thinking of everything he had told me. At night, I would remember how it all started and I do not know what he did to find that money, but he gave me the money to get the abortion.”

Her godfather gave her 3000 Córdobas (approximately USD112 at the time) and put her on a public bus, alone. He had arranged for her to receive the abortion from an older woman that practiced “natural medicine” in a nearby city. When Ana Maria arrived at the woman’s home, she was instructed to remove her pants and underwear and lie on a bed. Ana Maria did not receive any medication before the woman inserted a “device like the one used for a Papanicolau… and then another device like an iron rod” into her vagina.

After describing these devices, Ana Maria made a jerking motion back and forth with her arm to imitate the movement the woman used to perform the abortion.

Once it was over, the woman gave Ana Maria an injection of an unknown substance and told her that she would pass a few blood clots over the next few days. That night, however, Ana Maria’s condition worsened; she became feverish, felt disoriented, and began to pass dark, fetid clots of blood. She described the pain she experienced throughout the ordeal:

“I felt so much pain when they took her out of me. I felt pain when the blood was leaving my body and when I had the fever. I felt a terrible pain that only I suffered. I am [a] different [person] now because of those pains.”

Ana Maria was too afraid to tell her family about the assault or the abortion because she was uncertain how they would react. She was even more terrified of the potential legal repercussions that she could face for violating the total ban on abortion. Within a few days of the abortion, though, Ana Maria’s brother heard rumors of his sister’s situation from neighbors “in the street” and confronted her about what had happened. At first, Ana Maria denied that she had had an abortion, but her brother continued to ask for the truth. Though she was nervous, Ana Maria eventually told her brother everything that had happened – from her godfather’s incessant verbal harassment, to the rape, to the unsafe abortion she was forced to get.

Afraid for his sister’s life, Ana Maria’s brother contacted a local nurse who discreetly provides postabortion care (PAC) to women experiencing complications from unsafe abortion and other obstetric emergencies. This nurse is locally known to be one of the few health care providers who provide PAC despite many other providers’ fear of prosecution under the total ban. The nurse recommended that Ana Maria come to the hospital immediately.

Ana Maria spent almost two weeks as an inpatient at the only hospital in the region. She had become septic as a result of what she described as a “perforated uterus,” a common complication from unsafe abortion. [28] Upon her initial examination, the nurse was afraid that her uterus could not be repaired because the infection was so severe. Fortunately, the medical team administered an ultrasound, removed infected blood clots, and completed uterine surgery to repair the damage from the unsafe abortion. At the request of the gynecologist taking care of her, Ana Maria received the one-month contraceptive hormonal injection before being discharged. At the time of the interview, Ana Maria had not received the next month’s injection because she “didn’t have any use for a man.”

As a result of this experience, Ana Maria reported feelings of depression, isolation, and recurring dreams about a little girl, which she described in this way:

“After I was discharged, I always dreamt of a little girl and that she was mine, standing in my doorway and when I awoke, I couldn’t find her. I looked for her in my bed but she wasn’t there. And this has tormented me because, it’s true: I am the girl that committed this error, but the little girl was not at fault. He pressured me so strongly to get the abortion, so I did.”

Ana Maria had the same recurring dream every night for more than two weeks and she continued to feel depressed weeks after leaving the hospital. One of the sources of her depression was the isolation she felt because there was no one with whom she could share this experience.

According to Ana Maria, she longs to have other people to talk to about her experience – particularly those who may have had similar experiences. She also expressed a desire to pursue a law degree so that she can have a career in local government.

## Discussion and conclusions

Ana Maria’s case provides insight into the contextual factors effecting her ability to realize her sexual and reproductive health and rights in Nicaragua where restrictive legal policies and conservative cultural norms around sexuality abound. These contextual risk factors include social norms related to sexual health, laws targeting VAW, and the criminalization of abortion.

### Social norms related to sexual health

The fundamental relationship between structural inequality and sexual and reproductive rights has been duly noted; gender inequality, in particular, must be addressed in order to fulfill sexual rights for women. [29] As in many cases in Nicaragua, the fact that Ana Maria’s first sexual experience was nonconsensual and was initiated by an older male and trusted family friend highlights the uneven power relations between men and women in Nicaraguan culture, which propagate high instances of VAW and sexual assault. In a patriarchal society where *machismo* and gender inequality run rampant, women’s sexuality is further constrained by the stigmatization of sexual health and a culture of violence that limits women’s autonomy. The compound stigma surrounding sexual health in general, and rape in particular, negatively impacted Ana Maria’s knowledge and ability to access mental health and SRH services, including emergency contraception and post-rape care, which may have assisted her immediately following her assault. Before her brother intervened, Ana Maria’s fear of judgment and legal repercussions also prevented her from seeking PAC, which was necessary to save her life.

Comprehensive sexual education is a primary way to challenge these social norms and widespread stigma surrounding sexuality and SRH services, such as contraception and PAC, at the population level. Such education might have mitigated Ana Maria’s experience of unintended pregnancy through the provision of advance knowledge of emergency contraception and medical options in the event of pregnancy. CEDAW has recognized this missed opportunity for public health intervention in Nicaragua, and recommends sexual education as a means of addressing stigma related to sexuality, decreasing unintended pregnancy, and increasing the acceptability and use of family planning services throughout the country. [21] Furthermore, the lack of adolescent-friendly sexual education and SRH services symbolizes a social reluctance to acknowledge the reality that young people have sex. [30] Such ignorance results in a lack of information on healthy relationships and human reproduction, as well as experiences of unintended pregnancy, early motherhood, and unsafe abortion. Exposure to this type of information may have improved Ana Maria’s ability to protect herself, mitigated the impact of Nicaragua’s pervasive misogyny on her decision making, and lessened the influence of her godfather’s coercion before her experiences of rape and unsafe abortion.

### Individual and structural violence against women

Though we do not know explicit details of Ana Maria’s rape, the act of rape is inherently violent. The assault violated her right to enjoy sexual experiences free from coercion and violence. [3] To further constrain her sexual and reproductive rights, Ana Maria’s experience of rape resulted in an unintended pregnancy and an unsafe abortion that she was pressured into undergoing. Along with physical sequelae as a result of the procedure, she also expressed feelings of depression and isolation, which are common symptoms of post-traumatic stress disorder (PTSD). [31] These mental health consequences are forms of emotional violence that Ana Maria continued to experience long after the initial insult of physical violence. We can’t distinguish whether her mental health symptoms were a pre-existing condition or a result of the traumatic experience presented here. It is likely, however, that all parts of this experience impacted her mental and physical health. As reported elsewhere, perceived social criticism and a lack of social support are barriers to the fulfillment of sexual and reproductive health among young Nicaraguan women. [18] These contextual risk factors undoubtedly played a role in Ana Maria’s ability to navigate the circumstances surrounding her assault and its aftermath.

What legal recourse was feasibly available to Ana Maria for the crime of her sexual assault? To our knowledge, Ana Maria did not report the rape to authorities nor did her godfather ever face criminal charges for his actions. Yet Ana Maria’s own fear of prosecution for undergoing the unsafe abortion, as well as shame and fear of being stigmatized by others in her community, strongly influenced her decision not to report the rape -- even though Law 779 contains sanctions specific to those who commit rape.

In the event she had reported the crime, however, it is unclear if Law 779 would have provided justice. There are no data to suggest that Law 779 has led to an increase in the reporting or prosecution of rape at the national level. To the contrary, qualitative work in Nicaragua found a perceived increase in VAW following the passage of the law. [14] In Nicaragua, the inconsistent or ineffective enforcement of Law 779 is another factor worthy of consideration in cases like Ana Maria’s where individuals do not report such crimes. Documents like the UN Women Model Protocol have recently been released to improve the enforcement of laws like Law 779 in Latin American countries, presenting an opportunity for the effective operationalization of the law in Nicaragua. [32] If Law 779 is not adequately enforced, women like Ana Maria face the potential for re-victimization through the structural violence of impuity and continued exposure to VAW. To our knowledge, Ana Maria’s perpetrator faced no consequences for his perpetration of harassment, coercion and rape of Ana Maria. Moreover, in countries where abortion is criminalized, such as El Salvador, it is most often women who face criminal sanctions. [33] Indeed, it was Ana Maria herself who bore the physical and mental burden that resulted from her assault, unintended pregnancy, and unsafe abortion.

### The criminalization of abortion

The criminalization of health services is a strategy that governments use to regulate people’s sexuality and sexual activity. [34] The criminalization of services such as abortion limits women’s ability to make autonomous decisions about their SRH. By definition, laws that restrict access to health services exclude people from receiving the information and services necessary to realize the highest level of SRH possible. [5] The criminalization of abortion puts the health and well-being of individuals and communities at risk. Beyond the individual level, complications from unsafe abortion often put unnecessary and immeasurable financial burdens on health systems that are already stretched [28].

Ana Maria did not have a choice when it came to her abortion; the man who raped her coerced her to undergo an unsafe and illegal procedure. The criminalization of abortion in Nicaragua put Ana Maria’s health at risk in two ways: first, it prevented her from obtaining a safe abortion and second, it limited her access to comprehensive sexual health information that could have helped her address her unintended pregnancy, through emergency contraception. After the unsafe abortion procedure, her access to PAC was likely constrained by her own fear of the possible legal repercussions of undergoing an abortion, and was compounded by her inability to trust that a health care provider would maintain patient confidentiality and provide adequate PAC.

In Nicaragua, the total ban on abortion directly contradicts strategic objectives outlined in the Beijing Declaration, which guarantees women’s rights to comprehensive SRH care, including family planning and PAC services. Though providing PAC is not considered illegal under the total ban, many Nicaraguan health care providers refuse to treat women who have had unsafe abortions, which results in a ‘chilling effect’; providers do not want to be accused of being complicit in providing abortions so they refuse to provide PAC services. The ‘chilling effect’ put Ana Maria at risk of morbidity or mortality as a result of the complications that resulted from her unsafe abortion.

Equally troubling is the use of criminal law against individuals like Ana Maria as well as health care professionals that provide PAC. By requiring health care providers to report to the police women who have had abortions, the total ban violates the privacy inherent in the patient-provider relationship. Health care providers are faced with a dual loyalty to both the State’s laws and the confidentiality of their patients, which makes it difficult for providers to fulfill their professional obligations. It also makes health care professionals complicit in a discriminatory practice, one where women face legal sanctions in ways that men do not. The criminalization of abortion in Nicaragua therefore resulted in the fear, stigma, discrimination, and negative health outcomes observed in Ana Maria’s case.

The contextual risk factors that contributed to Ana Maria’s experience of rape, unintended pregnancy, and unsafe abortion are as follows: sexual assault, impunity for violence, gender inequality, restrictive social norms around SRH, stigma resulting from unintended pregnancy and abortion, harmful health impacts from an unsafe abortion, and fear of prosecution due to the total ban. Her first sexual experience was forced and nonconsensual and preceded by months of harassment. Social norms made taboo any discussion of the harassment and sexual violence she experienced at the hands of her godfather; without social support, she was coerced into undergoing an unsafe abortion that resulted in serious mental and physical health sequelae. The illegal nature of abortion in Nicaragua placed Ana Maria at risk for social stigma as well as criminal prosecution. Her subsequent underutilization of family planning services at the time of the interview also placed Ana Maria at risk for an unintended pregnancy in the future; other long-term physical and mental health effects of her experience remain unknown.

The realization of one’s sexual and reproductive rights guarantees autonomous decision-making over one’s fertility and sexual experiences. However, Ana Maria’s story demonstrates that an individual’s SRH decisions are not made in isolation, free from the influence of social norms and national laws. Far too many women experience their sexuality in the context of individual and structural violence, such as VAW and gender inequality. This case highlights the contextual risk factors that contributed to Ana Maria’s experience of violence, unintended pregnancy, and unsafe abortion; we must continue to critically investigate these factors to ensure that experiences like Ana Maria’s do not become further normalized in Nicaragua. Due to restrictive social norms around SRH, Ana Maria grew up experiencing stigma and taboo associated with sex, sexuality, contraceptive use and abortion. She also lacked access to information regarding SRH, healthy relationships, and how to respond to VAW before she was assaulted. After her assault, she did not have access to post-rape care, emergency contraception, safe abortion services, or mental health services to help her process this trauma. Shame and fear of stigma also prevented Ana Maria from reaching out for social support from family, friends, or the health or legal system. From the legal perspective, inadequate enforcement of VAW laws and the criminalization of abortion further exacerbated the trauma Ana Maria experienced.

It would require active engagement from the Nicaraguan government to address the contextual risk factors identified herein to protect their citizens’ right to health and prevent future experiences like Ana Maria’s. These efforts are particularly relevant given recent political unrest throughout Nicaragua including anti-government protests demanding the president’s resignation. [35] Nicaraguans’ right to health is at risk not only due to the widespread violence, but also because health care workers are being dismissed and persecuted nationwide. [36] Sexual and reproductive health researchers, advocates, and the public will continue to monitor Nicaragua’s response to the immediate demands and needs of its citizens -- including the demand that Nicaraguan women like Ana Maria are able to fully exercise their sexual and reproductive rights in times of both conflict and peace.

## Additional file

Additional file 1: Interview Guide. (ZIP 32 kb)

## Data Availability

Deidentified data are available upon reasonable request.

## Abbreviations

CCPR: Committee on Civil and Political Rights
CEDAW: Committee on the Elimination of all forms of Discrimination Against Women
IDIs: In-Depth Interviews
PAC: Postabortion Care
PTSD: Post-Traumatic Stress Disorder
SES: Socioeconomic Status
SRH: Sexual and Reproductive Health
UN: United Nations
VAW: Violence Against Women
WHO: World Health Organization

## References

[1] United Nations Population Fund (UNFPA). Report of the international conference on population and development. Cairo; 1994. Available from: http://www.un.org/popin/icpd/conference/offeng/poa.html.

[2] United Nations (UN). Fourth world conference on women: Beijing declaration and platform for action. Beijing; 1995. Available from: http://www.un.org/en/events/pastevents/pdfs/Beijing_Declaration_and_Platform_for_Action.pdf.

[3] World Health Organization (WHO). Sexual health, human rights and the law. 2015; Available from: http://apps.who.int/iris/bitstream/10665/175556/1/9789241564984_eng.pdf?ua=1

[4] United Nations (UN). Convention on the elimination of all forms of discrimination against women (CEDAW). A/RES/34/180. 1979. Available from: https://www.ohchr.org/EN/ProfessionalInterest/Pages/CEDAW.aspx

[5] United Nations (UN). Substantive issues arising in the implementation of the International Covenant on Economic, Social, and Cultural Rights: General comment no. 14. E/C.12/2000/4. 2000. Available from: http://docstore.ohchr.org/SelfServices/FilesHandler.ashx?enc=4slQ6QSmlBEDzFEovLCuW1AVC1NkPsgUedPlF1vfPMJ2c7ey6PAz2qaojTzDJmC0y%2b9t%2bsAtGDNzdEqA6SuP2r0w%2f6sVBGTpvTSCbiOr4XVFTqhQY65auTFbQRPWNDxL

[6] Carcedo A. (2008). Femicide in Central America 2000–2006. In strengthening understanding of femicide: Using research to galvanize action and accountability (p. 7–25). Program for Appropriate Technology in Health (PATH), InterCambios, Medical Research Council of South Africa (MRC), and World Health Organization (WHO) Meeting in Washington, DC, April 2008.

[7] Sternberg P (2000). Challenging machismo: promoting sexual and reproductive health with Nicaraguan men. Gend Dev.

[8] Sternberg P, White A, Hubley JH (2007). Damned if they do, damned if they don’t: tensions in Nicaraguan masculinities as barriers to sexual and reproductive health promotion. Men Masculinities.

[9] Arciniega GM, Anderson TC, Tovar-Blank ZG, Tracey TJG (2008). Toward a fuller conception of machismo: development of a traditional machismo and caballerismo scale. J Couns Psychol.

[10] Torres VirgilioMariano Salazar, Goicolea Isabel, Edin Kerstin, Öhman Ann (2012). ‘Expanding your mind’: the process of constructing gender-equitable masculinities in young Nicaraguan men participating in reproductive health or gender training programs. Global Health Action.

[11] National Institute for Development Information (INIDE). Nicaraguan Demographic and Health Survey 2006/07: Final Report. Managua: Nicaragua. 2008. Available from: http://www.inide.gob.ni/endesa/Endesa_2006/Endesaingles.pdf.

[12] 4 April (2013). United nations (UN) women. Femicide in Latin America.

[13] National Assembly, Nicaragua. Law 779: The Comprehensive Law Against Violence Against Women and Reforms to Law No. 641, “Penal Code.” Managua, Nicaragua. 2012. Available from: https://www.poderjudicial.gob.ni/pjupload/leyes/Ley_No_779_Ley_Integral_Contra_la_Violencia_hacia_la_Mujer.pdf

[14] Luffy SM, Evans DP (2015). Rochat RW. “It is better if I kill her”: perceptions and opinions of violence against women and femicide in Ocotal, Nicaragua after law 779. Violence Gend.

[15] Amnesty International (2010). Nicaragua: listen to their voices and act. Stop the rape and sexual abuse of girls in Nicaragua.

[16] World Health Organization (WHO), London School of Hygiene and Tropical Medicine, South African Research Council. Global and regional estimates of violence against women: prevalence and health effects of intimate partner violence and non-partner sexual violence against women. Geneva: WHO; 2013. Available from: http://www.who.int/reproductivehealth/publications/violence/9789241564625/en/

[17] World Health Organization (WHO). Safe abortion: technical and policy guidelines for health systems – 2nd ed. 2012. Available from: http://apps.who.int/iris/bitstream/10665/70914/1/9789241548434_eng.pdf 23700650

[18] Luffy SM, Evans DP, Rochat RW (2015). “Siempre me critican”: barriers to reproductive health in Ocotal, Nicaragua. Rev Panam Salud Publica.

[19] Berglund S, Liljestrand J, Marin FM, Salgado N, Zelaya E (1997). The background of adolescent pregnancies in Nicaragua: a qualitative approach. Soc Sci Med.

[20] Walsh J, Mollmann M, Heimburger A (2008). Abortion and human rights: examples from Latin America. IDS Bulletin, Institute of Development Studies.

[21] United Nations (UN). Concluding comments of the Committee on the Elimination of Discrimination against Women: Nicaragua. CEDAW/C/NIC/CO/6. 2007. Available from: http://docstore.ohchr.org/SelfServices/FilesHandler.ashx?enc=6QkG1d%2fPPRiCAqhKb7yhsqMFgv33OTgoZv7ZAgL6thDRNHOIdSmvBad%2f8i4XoKe2V5DyBrEEI%2bsOdccm877lZ2zUTTB3%2blqL93FUU1suHxkCT5dGDpWG1VxMxMULVrjx

[22] Lion KC, Prata N, Stewart C (2009). Adolescent childbearing in Nicaragua: a quantitative assessment of associated factors. Int Perspect Sex Reprod Health.

[23] Reuterswärd C, Zetterberg P, Thapar-Björkert S, Molyneux M (2011). Abortion law reforms in Colombia and Nicaragua: issue networks and opportunity contexts. Dev Chang.

[24] UN Human Rights Committee (HRC), CCPR General Comment No. 28: Article 3 (The Equality of Rights Between Men and Women). 2000 Mar, CCPR/C/21/Rev.1/Add.10. Available from: https://tbinternet.ohchr.org/Treaties/CCPR/Shared%20Documents/1_Global/CCPR_C_21_Rev-1_Add-10_6619_E.pdf

[25] Barot S (2011). Unsafe abortion: the missing link in global efforts to improve maternal health. *Guttmacher Policy Review*. Spring.

[26] Say L, Chou D, Gemmill A, Tunçalp O, Moller A, Daniels J, Gülmezoglu AM, Temmermann M, Alkema L (2014). Global causes of maternal death: a WHO systematic analysis. Lancet Global Health.

[27] Miller AM, Roseman MJ (2011). Sexual and reproductive rights in the United Nations: frustration or fulfillment?. Reproductive Health Matters.

[28] Haddad LB, Nour NM (2009). Unsafe abortion: unnecessary maternal mortality. Rev Obstet Gynecol.

[29] Yamin AE, Boulanger VM (2013). Embedding sexual and reproductive rights in a transformational development framework: lessons learned from the MDG targets and indicators. Reproductive Health Matters.

[30] Mirembe F, Karanja J, Hassan EO, Faundes A (2010). Goals and activities proposed by countries in seven regions of the world toward prevention of unsafe abortion. Int J Gynecol Obstet.

[31] Tinglof S, Hogberg U, Lundell IW, Svanberg AS (2015). Exposure to violence among women with unwanted pregnancies and the association with post-traumatic stress disorder, symptoms of anxiety and depression. Sexual & Reproductive HealthCare.

[32] Villa Quintana CR. Modelo de protocolo latinoamericano de investigación de las muertes violentas de mujeres por razones de género (femicidio/feminicidio). 2014. Accessed from: http://www.unwomen.org/-/media/headquarters/attachments/sections/library/publications/2014/modelo%20de%20protocolo.ashx?la=es

[33] Viterna J, Guardado Bautista JS (2017). Pregnancy and the 40-year prison sentence: how “abortion is murder” became institutionalized in the Salvadoran judicial system. Health Hum Rights.

[34] Gruskin S, Ferguson L (2009). Government regulation of sex and sexuality: in their own words. Reproductive Health Matters.

[35] McDonnell PJ. Here’s what you need to know about the crisis in Nicaragua. Los Angeles Times July. http://www.latimes.com/world/la-fg-nicaragua-unrest-20180726-story.html

[36] Hanson L. Side effects: persecution of health workers in Nicaragua. Health and Human Rights Journal Blog. 2018; Available from: https://www.hhrjournal.org/2018/08/side-effects-persecution-of-health-workers-in-nicaragua/?platform=hootsuite.

