# Supplementary material for: “Regardless, you are not the first woman”: an illustrative case study of contextual risk factors impacting sexual and reproductive health and rights in Nicaragua
Source: BMC Womens Health. 2019 Jun 14;19:76. doi: 10.1186/s12905-019-0771-9 (PMC6570882; doi:10.1186/s12905-019-0771-9)
Supplement: Supplementary file 1 — Interview Guide. (ZIP 32 kb) [file 12905_2019_771_MOESM1_ESM.zip › Luffyetal_IDI Guide_English05.28.19R7.docx]

**In-depth Interview Guide**

Introductory questions:

- How old are you?
- Are you legally married?
- Who do you live with?
- Do you have a job?
- Did you go to high school?
- Are you religious / Catholic?

Pregnancy history:

- How many times have you been pregnant?
  - How old were you?
- How many children/births have you had?
  - Did you have any complications?
- When was your last pregnancy?
- Were you using a method of family planning before you became pregnant?
- Before your last pregnancy, did you want more children?
  - Probes: never, later, now
- When you became pregnant, did you want to become pregnant at that time?

Unintended pregnancy:

- Have you had a pregnancy that was unintended?
- How did your partner/husband react?
- How did your family react?
- Why do you think they reacted the way they did?
- How did you feel when you found out you were pregnant?
  - Probes: nervous, happy, sad, depressed, worried
  - Or: What were your first thoughts when you found out you were pregnant?
- Did you want to continue the pregnancy?
  - If you did not want to continue the pregnancy, what did you see as your options to end it?
- What was the result of the pregnancy?
  - Probes: a birth, miscarriage, abortion
- How did you come to this decision?
- Was there a family member or friend that emotionally helped you during this time?
- How did your life change after you gave birth?
  - Do you want more children at this time? Are you using a method of family planning now?

Reproductive heath care:

- How did you feel when you went to the doctor’s office during your pregnancy?
  - Probes: scared, nervous, at ease, uncomfortable
- How did the medical staff treat you during your appointments?
  - How did they treat you when you were in labor?
- Did you get the kind of attention/care you wanted?
- Did you receive information about methods of family planning after you gave birth?
  - If so, which methods?
- Did you use a method of family planning after giving birth?
  - If so, which method did you use?

Unintended pregnancy in general:

- How common are unintended pregnancies here in [city]?
  - Why?
- The percentage of unintended pregnancies in León is 18% and 9.5% in Managua. The percentage of unintended pregnancies here is 20%.
  - Why do you think the percentage is higher here?
