# Supplementary material for: “Regardless, you are not the first woman”: an illustrative case study of contextual risk factors impacting sexual and reproductive health and rights in Nicaragua
Source: BMC Womens Health. 2019 Jun 14;19:76. doi: 10.1186/s12905-019-0771-9 (PMC6570882; doi:10.1186/s12905-019-0771-9)
Supplement: Supplementary file 1 — Interview Guide. (ZIP 32 kb) [file 12905_2019_771_MOESM1_ESM.zip › Luffyetal_IDI Guide_Spanish05.28.19R7.docx]

**Guía de Entrevista en Profundidad**

Preguntas Introductorias:

- ¿Cuántos años tiene usted?
- ¿Esta legalmente casada?
- ¿Usted con quien vive?
- ¿Usted tiene trabajo?
- ¿Usted estudio bachillerato/ secundaria en el colegio?
- ¿Es usted católica religiosa?

Historial de embarazos:

- ¿Cuántos veces ha quedado en estado de embarazo?
  - ¿Cuántos años tenía?
- ¿Cuántos niños/partos ha tenido?
  - ¿Había complicaciones?
- ¿Cuándo fue su ultimo embarazo?
- ¿Estaba planificando antes que salió embarazada?
- Antes de su ultimo embarazo, ¿usted quería más hijos?
  - Pruebas: nunca, más tarde, ahora
- ¿Cuándo salió embarazada, quería estar embarazada ya?

Embarazo no deseado:

- ¿Ha tenido algún embarazo no deseado?
- ¿Como reaccionó su pareja/esposo?
- ¿Como reaccionó su familia?
- ¿Por qué ellos reaccionaron como así?
- ¿Como se sintió cuando se entero que estaba embarazada?
  - Pruebas: Nerviosa, feliz, triste, deprimida, preocupada
  - Ósea, ¿cual eran sus primeros pensamientos cuando se entero que estaba embarazada?
- ¿Usted quiso continuar el embarazo?
  - Si usted no quería continuar el embarazo, ¿cuales opciones veía usted para terminar el embarazo?
- ¿Cual fue el resultando del embarazo?
  - Un parto, aborto involuntario, aborto provocado
- ¿Como llego a esta decisión?
  - ¿Qué cosas tenían influencia en su decisión?
- ¿Hubo una persona familiar o un amigo/una amiga que la apoyó emocionalmente?
- ¿En que cambiado su vida después de su parto?
  - ¿Ahora quiere más hijos? ¿Estás planificando?

Servicios de la salud reproductiva:

- ¿Como se sintió cuando usted fue al consultorio medico durante su embarazo?
  - Pruebas: asustada, nerviosa, cómoda, incomoda
- ¿Como la trataron los médicos durante sus sitas?
  - ¿Y durante el parto?
- ¿Recibió el tipo de atención/cuidado que usted quería?
- ¿Como cambiaria usted la manera en que la trataron?
- ¿Recibió información sobre métodos de planificación familiar después del parto?
  - ¿Cuál métodos?
- ¿Usó un método de planificación después del parto?
  - ¿Cuál método para planificar usó?

Embarazos no deseados en general:

- ¿Qué tan común son los embarazos no deseados aquí en [la ciudad]?
  - ¿Por qué?
- Los porcentajes de embarazos no deseados en León son 18%, y 9.5% en Managua. El porcentaje de embarazos no deseados aquí es 20%.
  - ¿Por qué piensas que esa es la situación aquí?
